# Diagnostic Approach for Monitoring Hydroclimatic Conditions Related to Emergence of West Nile Virus in West Virginia

**DOI:** 10.3389/fpubh.2015.00010

**Published:** 2015-02-12

**Authors:** Antarpreet Jutla, Anwar Huq, Rita R. Colwell

**Affiliations:** ^1^Department of Civil and Environmental Engineering, West Virginia University, Morgantown, WV, USA; ^2^Maryland Pathogen Research Institute, University of Maryland, College Park, MD, USA; ^3^Center for Bioinformatics and Computational Biology, University of Maryland, College Park, MD, USA

**Keywords:** vector-borne disease, precipitation, land surface temperature, MODIS, infectious diseases, prediction models

## Abstract

West Nile virus (WNV), mosquito-borne and water-based disease, is increasingly a global threat to public health. Since its appearance in the northeastern United States in 1999, WNV has since been reported in several states in the continental United States. The objective of this study is to highlight role of hydroclimatic processes estimated through satellite sensors in capturing conditions for emergence of the vectors in historically disease free regions. We tested the hypothesis that an increase in surface temperature, in combination with intensification of vegetation, and enhanced precipitation, lead to conditions favorable for vector (mosquito) growth. Analysis of land surface temperature (LST) pattern shows that temperature values >16°C, with heavy precipitation, may lead to abundance of the mosquito population. This hypothesis was tested in West Virginia where a sudden epidemic of WNV infection was reported in 2012. Our results emphasize the value of hydroclimatic processes estimated by satellite remote sensing, as well as continued environmental surveillance of mosquitoes, because when a vector-borne infection like WNV is discovered in contiguous regions, the risk of spread of WNV mosquitoes increase at points where appropriate hydroclimatic processes intersect with the vector niche.

## Introduction

West Nile virus (WNV) is a mosquito-transmitted *Flavivirus* belonging to the Japanese encephalitis antigenic complex of the family *Flaviviridae*. The natural transmission cycle for WNV is usually limited to birds and mosquitoes, routing to a host, which may be birds, humans, or other mammals and reptiles, all of which become infected when bitten by mosquitoes (1). Since its appearance in the northeastern United States in 1999, WNV was reported throughout the entire continental United States in a relatively short period of time. In fact, WNV is now a very serious vector-borne disease in the United States because of its high morbidity rate in humans, with severe impact on avian populations (2). Infected mosquitoes, in general, survive in hot, humid environments where warm temperatures prevail and the vegetation is dense (3). The functional repertoire of a causative agent of mosquito-based disease is unusually broad, accommodating two distinctively different environments: the micro-environment of the vector and the macro-environment of the aquatic habitat. In the study reported here, the micro-environment is defined as comprising those processes within the mosquito (vector), while the macro-environment refers to hydrological, ecological, and climatic processes affecting growth and proliferation of the mosquito. Micro-environmental understanding of the disease is essential if vaccine or treatment protocols are to be maximally effective. However, a single disciplinary approach will have limitations for prediction considering mosquitoes adapt to their environment. Furthermore, new and adaptive biotypes emerge over time, making it unlikely that mosquitoes can be eradicated.

Remote sensing and geographic information systems (GIS) have proven to be useful tools for gathering information helpful in understanding relationships between large-scale hydroclimatic and epidemiological processes that provide risk assessment for human infections (4). Remotely sensed data in the visible and infrared spectra used to map and forecast vector/water-borne disease at spatial scales ranging from landscapes to the entire earth (2).

The U.S Centers for Disease Control reported in August, 2012 that 1,118 WNV cases and 41 deaths had been confirmed nation-wide. Because WNV is a relatively new disease in the continental USA, only a few of studies have been published and these focus primarily on single outbreak at discrete locations. Employing hydroclimatic processes and variables, e.g., land surface temperature (LST), normalized difference vegetation index (NDVI), and actual evapotranspiration (ETa), all derived from Moderate Resolution Imaging Spectroradiometer (MODIS) over the Great Plains, and linked it with outbreaks of WNV (2). Table 1 summarizes key information from some of the current literature on association of hydroclimatic variables, and WNV. Two important observations were derived from the table is that there is emerging consensus on a triggering mechanism relating hydroclimatic conditions to mosquito abundance and human cases. However, some of the findings from the studies are disputed in literature, e.g., Liu and Weng (1) reported that NDVI were not a significant factor for WNV transmission in Los Angeles, CA, USA Chuang et al. (2), on the other hand, developed regression models with NDVI as one of the major explanatory variables over North Great Plains. A parsimonious hypothesis on triggering mechanisms for WNV is perhaps lacking, due primarily to absence of data on the disease, as well as snapshot types of analysis of individual outbreaks occurring at discrete locations.

**Table 1:** **Summary of key available remote sensing based WNV studies on hydroclimatic processes in the continental United States**.

	Author	Reference	Associated variables	Geographic region
1	Zou et al. (2006)	(5)	Water bodies, vegetation	Wyoming, USA
2	Liu et al. (2008)	(6)	Total length of streams, size of wetlands	Indianapolis, USA
3	Liu et al. (2011)	(7)	Vegetation, precipitation	Virginia, USA
4	Cleckner et al. (2011)	(8)	Vegetation, water bodies	Virginia, USA
5	Liu and Weng (2009)	(9)	Land cover, surface temperature	Chicago, USA
6	Chuang and Wimberly (2012)	(2)	ET, vegetation, surface temperature	Great Plains, USA
7	Liu and Weng (2012b)	(10)	Summer temperature, deviation of temperature, vegetation, elevation, vegetation	Southern California, USA
8	Chuang et al. (2012)	(11)	Air temperature, vegetation density	South Dakota, USA
9	Liu and Weng (2012a)	(1)	Elevation, urban land cover	Los Angeles, USA

West Nile virus may never be eradicated since the disease vector is naturally present in the environment. With no vaccine available, a new approach for prediction and prevention of the disease is needed. The aim of this short communique is to demonstrate suitability of hydroclimatic processes derived from remote sensing data to identify a region(s) where human WNV cases are not yet epidemic or very few and unnoticed during the past years. Within this context, the objective is to understand association of two large-scale geophysical processes, LST and precipitation with abundance of WNV mosquitoes in an area where human infection is not yet epidemic. The hypothesis that increase in temperature in conjunction with abundant vegetation lead to increase in mosquito activity, following precipitation was tested, with an alternate hypothesis that a stable relationship between a given geophysical variable and mosquito abundance does not exist, making satellite monitoring not feasible for prediction of WNV.

## Data

**Figure 1:**
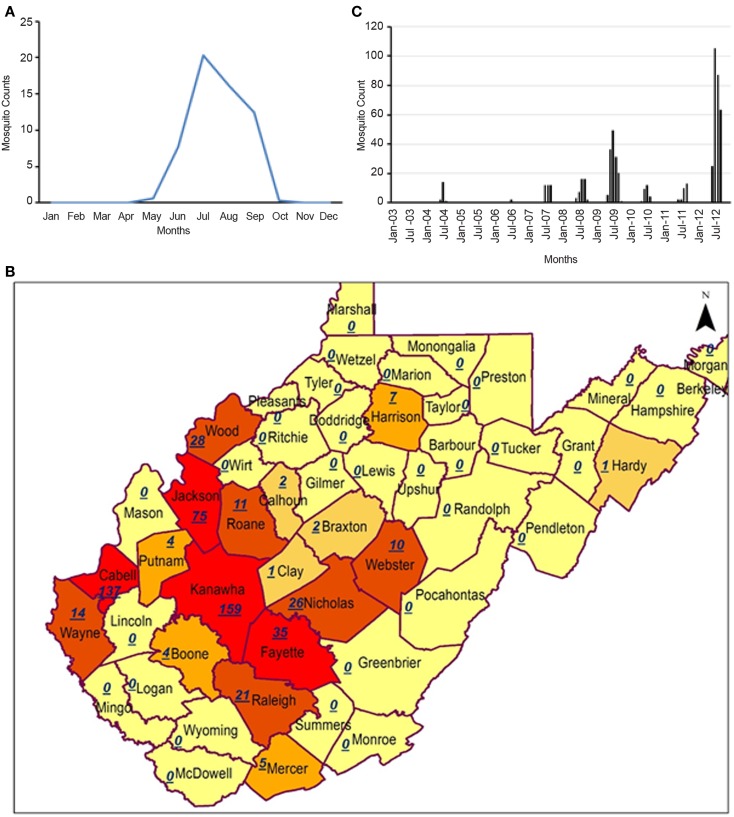
**(A)** Seasonality (monthly average) of West Nile Virus positive mosquitoes from 2003 to 2012; **(B)** cumulative spatial distribution of positive infectious mosquitoes from 2003 to 2012 [red color indicate positive WNV mosquito counts >9; orange color indicates counts between 1 and 9; and yellow indicates no reported positive mosquito count]; and **(C)** time series for infectious mosquito counts for West Virginia in last 10 years (data from ArboNET).

West Virginia was selected as the region of interest for the study because of the relatively low number of reported WNV human cases, but a steady increase in the number of mosquitoes testing positive for WNV. WNV mosquito activity has distinctive seasonality, generally occurring during the early autumn season (Figure 1A), with the potential to be linked with hydroclimatic variability. A sudden increase in WNV mosquitoes during 2012 (12) in this historically virus free region, combined with low prevalence of WNV cases in West Virginia, as occurred in, e.g., Colorado, California, and Texas, provided an opportunity for a case study in the region. Most of the WNV positive mosquitoes collected from pools in West Virginia were reported from June through September (Figure 1A), suggestive of a seasonal geophysical changes in the environment affecting the mosquitoes (8). The first cases of human WNV in West Virginia occurred in 2002. Weekly data on mosquitoes testing positive for West Nile were obtained from the Centers for Disease Control and Prevention (CDC) ArboNET database, a national surveillance system for arboviral diseases in the United States. Satellite data were acquired from the MODIS and reprojected using MODIS reprojection tool over the entire West Virginia (rectangular upper left corner 40°N and 82.67°W and lower right corner 37.17°N and 78.15°W). LST (MOD11A2) data were obtained in an 8-day composite to minimize cloud effects and the fact that our analysis is on monthly scale. Precipitation data obtained from the National Oceanic and Atmospheric Administration, National Weather Service. Precipitation data (PRECL) for the same grid box, available at 1° × 1° resolution, were obtained from National Oceanographic and Atmospheric Administration (NOAA).

## Results and Discussions

### Empirical observation analysis

Geographically, WNV positive mosquitoes were reported in central to southern West Virginia (Figure 1B). Interestingly, these are regions including Kanawha County that are economically important to the state, where with coal, chemical, and natural gas industries are located. However, few human cases are reported for the entire state of West Virginia, mainly because of the low population density of West Virginia even in the urban areas. Figure 1A shows that mosquitoes have a distinct seasonality, with peak activity during the summer (highest observed positive cases in the month of July), but the data are limited since sampling was not conducted during the winter, the assumption being that mosquitoes are not active in the environment in cold weather (12). Monthly time series of WNV positive mosquitoes shows that mosquito activity is steadily increasing each year (Figure 1C). Years 2012 (high number of WNV positive mosquitoes) and 2011 (low number of WNV positive mosquitoes) were selected for the analysis. In total, 37% of the pools tested yielded WNV positive *Culex restuans* and *Culex pipiens* (68% of all mosquitoes collected during 2012), the primary WNV species in the region (12). Two adjacent years were selected for study to quantify relative changes in large-scale hydroclimatic processes.

**Figure 2:**
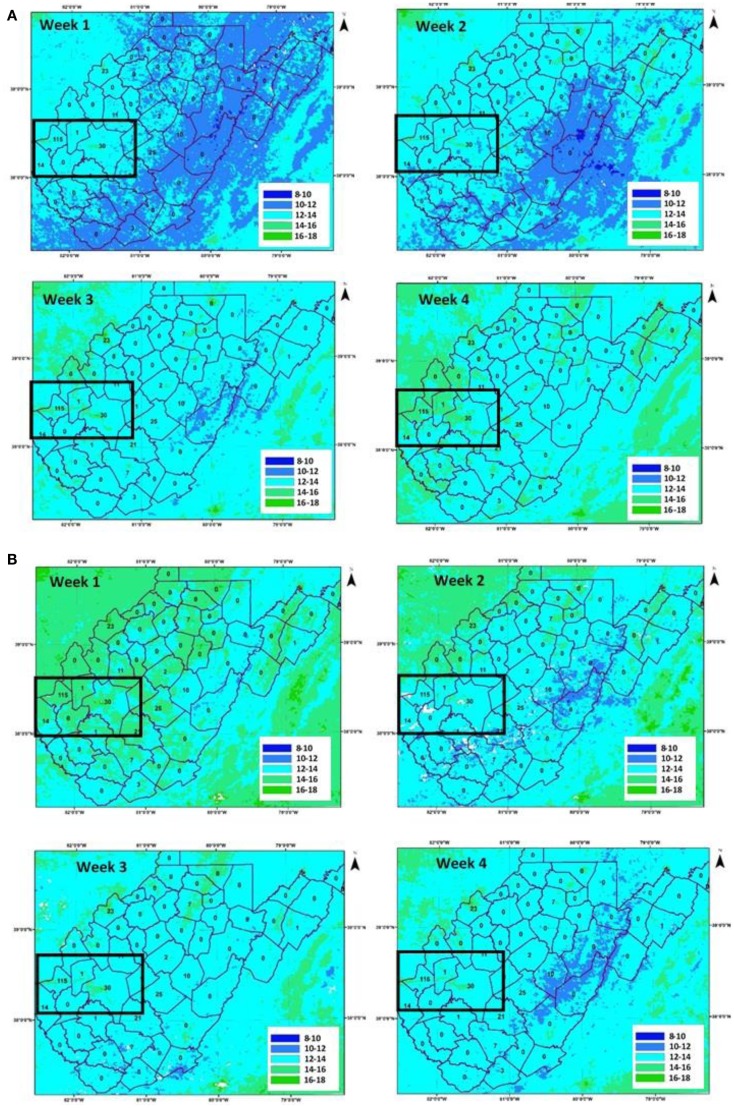
**Land surface temperature (°C) for (A) June 2012 and (B) July 2012**. (Black box in the figure – 38.6825°N to 37.8788°N; 82.5844°W to 81.0955°W; and include Cabell and Kanawha counties of West Virginia).

**Figure 3:**
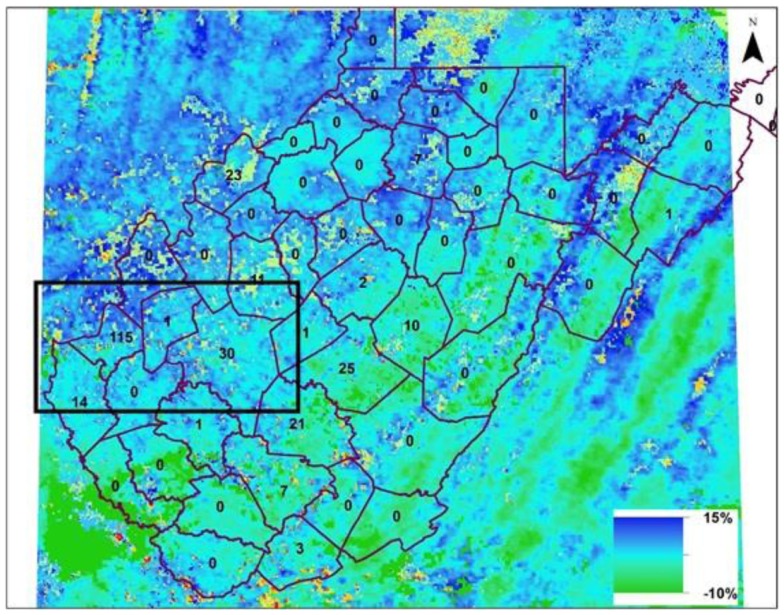
**Percentage change in LST in July 2012 with respect to July 2011**. The inset numbers on map are the percent difference in WNV positive mosquitoes during 2012 in counties of West Virginia compared to 2011. (Black box in the figure – 38.6825°N to 37.8788°N; 82.5844°W to 81.0955°; and include Cabell and Kanawha counties of West Virginia).

The 8-day composite LST from MODIS Terra platform was used instead of daily LST data to minimize missing values caused by cloud contamination. Figures 2A,B show eight panels for LST for June and July, respectively. In 2012, Cabell and Kanawha counties showed highest number of WNV positive mosquitoes (Figure 1B). If presence of mosquitoes were related to temperature, a steady increase in temperature should be observed and those regions of high temperatures show more WNV positive mosquitoes. Figure 2A (top left panel) shows distribution of LST (average areal temperature 10.5°C) during the first week of June, 2012. As land surface and air warm, the areal average increased (to 16.0°C) at the end of the fourth week of June. The temperature peaked during the first week of July to 17.5°C, falling sharply thereafter. Traditional time-series based correlation will not be applicable due to limited disease data as well as presence of zeroes in the time series. Two observations from Figure 2 include (i) the largest number of WNV positive mosquitoes were found in regions where a rapid increase in LST occurred (black box in the figure – 38.6825°N to 37.8788°N; 82.5844°W to 81.0955°W; and include Cabell and Kanawha counties of West Virginia) and (ii) the LST dropped within a few weeks in July, with spread of WNV positive mosquitoes limited to western counties of the region (Figure 2B). If the relationship between LST and warm air is valid, then a difference in inter-annual variability of LST should have been observed. Percent difference is shown in Figure 3, between monthly LST in July 2012 (highest number of WNV positive mosquitoes) and July 2011 (relative low number of WNV positive mosquitoes). Western counties (including Cabell and Kanawha, close to the Ohio and Elk rivers, respectively) experienced 10–15% increase in LST in July of 2012, providing complementary evidence that LST is an important hydroclimatic process related to emergence and spread of WNV positive mosquitoes. One may argue that the northeastern counties experienced a similar increase in temperature during 2012 without concurrent increase in the number of WNV positive mosquitoes. However, the maximum temperature recorded in the northeastern counties, which are at higher elevation than the western counties during 2012 was 10°C, compared to 17°C in the western counties (highlighted by the black box), providing a plausible explanation for the absence of mosquitoes in the region and corroborating thresholds for temperature effect on mosquito growth documented by other investigators (13). The counties adjacent to Cabell and Kanawha (e.g., Mason, Putnam, and Lincoln) show similar LST values with Cabell and Kanawha but the number of WNV positive mosquitoes of these counties are very low or 0, this is perhaps lack of environmental sampling in the region. On the other hand, several counties (e.g., Fayette, Nicholas, and Webster) show similar warming trend of LST with Cabell and Kanawha and hence have high number of WNV positive mosquitoes.

**Figure 4:**
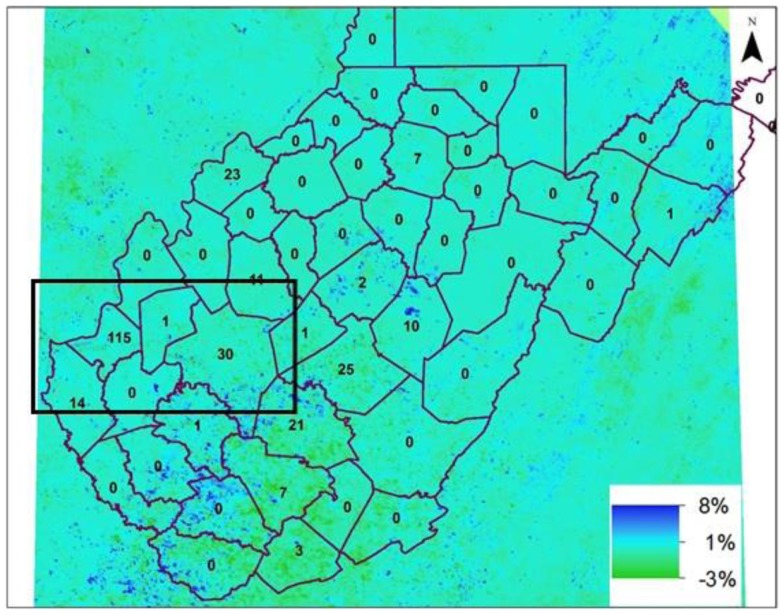
**Percentage change in NDVI in July 2012 with respect to July 2011**. The inset numbers on map are the WNV positive mosquitoes in year 2012 in counties of West Virginia. (Black box in the figure – 38.6825°N to 37.8788°N; 82.5844°W to 81.0955°; and include Cabell and Kanawha counties of West Virginia).

**Figure 5:**
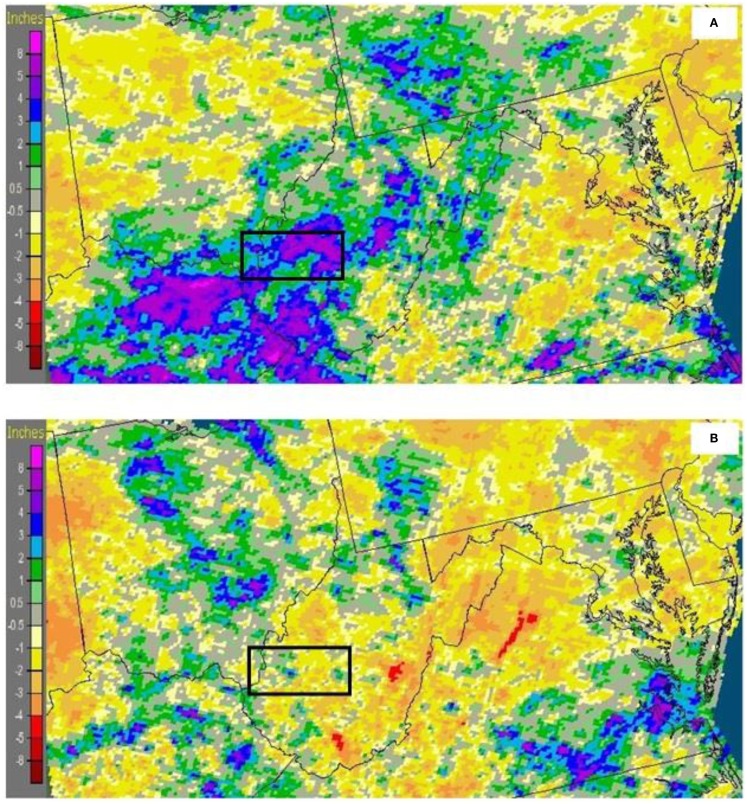
**Departure of precipitation from historical averages during (A) July 2012 and (B) July 2011**. Data and images were obtained from NOAA-National Weather Service. (Black box in the figure – 38.6825°N to 37.8788°N; 82.5844°W to 81.0955°; and include Cabell and Kanawha counties of West Virginia).

Increase in LST may not be sufficient to explain the expansion of mosquitoes throughout the region. Mosquitoes prefer a humid environment that often occurs in areas of high vegetation, since such areas store more volumetric water in the vadoze zone that is easily accessible, maintaining higher humidity. To assess the effects of vegetation, we used a 16-day NDVI product from the MODIS Terra platform. Percentage difference in NDVI values between July 2012 and 2011 were computed, with the premise that NDVI values should be higher in 2012 than during the preceding year. Figure 4 shows an average increase of 4.5% in NDVI, marked by the black box, where high prevalence of WNV positive mosquitoes was observed in 2012 (average NDVI 0.89). However, high values (NDVI > 0.89) of NDVI indicates that the region is already saturated with vegetation, which it covers part of Appalachian forests. Precipitation was above average for July 2012 (Figure 5A) and below average for 2011 (Figure 5B). Rainfall in July 2012 was 6″ above normal (black box) compared to July 2011, with 2″ less than normal precipitation, which may be a contributing factor to the increase in the NDVI in July 2012 and substantiating the hypothesis that increased temperature and followed by precipitation are associated with increase in mosquito population, namely *Cx. pipiens*/*restuans*, the primary WNV vector in the eastern United States (12, 14, 15).

### Model for understanding long-term association of WNV with hydroclimatic processes

Although empirical analysis supports the validity of the proposed hypothesis, however, such observations are based on one incidence of disease epidemic in West Virginia. In order to generalize the outcomes and perhaps develop a predictive ability to determine WNV mosquito risks, a suitable mathematical model is required. Therefore, we developed logistical regression models to understand association of LST and precipitation with WNV time series. It must emphasized that accurate number of diseased mosquitoes as well as human cases cannot be determined in a realistic sense; however, our interest is to identify hydroclimatic trigger conditions that may form a basis for a future forecasting system. Hence, as a complementary analysis, we used logistic regression model to understand the lead–lag relationships between positive WNV mosquitoes’ time series and LST as well as precipitation. For the development of the model, we averaged all pixels of LST in all counties, in Figure 1B, where more than nine positive mosquitoes (counties in red color) were reported for last 10 years. NOAA’s precipitation reconstruction over land dataset (available at 1° × 1° resolution) over the selected counties (Figure 1B) were used in the development of the model. Performance statistics of five models was shown in Table 2 where goodness of fit tests (Pearson and Hosmer–Lemeshow) indicate that the Model 3 (LST and precipitation at concurrent time period) performs the best among the rest of the models. The *p*-values of 0.75 (Hosmer–Lemeshow) and 0.64 (Pearson) for Model 3 shows that there is insufficient evidence to claim that the model does not fit the data. The statistical analysis was performed in Minitab in which null hypotheses corresponds to model adequately fitting the data. The *p*-values for Hosmer–Lemeshow and Pearson tests were >0.05 (statistical confidence at 95%), and hence does not reject null hypotheses. Goodman–Kruskal and Kendall Tau-a are the measures of association that can have a value between 0 and 1, higher the value, better the predictive ability (>50%) of the model. For Model 3 (α = 5.25; β = 0.68; γ = 0.36; all coefficients are statistically significant at *p* < 0.05), Goodman–Kruskal and Kendall Tau-a values of 0.65 and 0.60, respectively, were obtained and it indicates that model is likely to have predictive abilities for identifying risks of conditions for WNV mosquitoes in the region. Model 1 and Model 2 were developed to ascertain association of individual hydroclimatic process on WNV time series; however, both of the models failed to capture variability in the time series, further indicating that it is perhaps the compound effect of both LST and precipitation that results in mosquito abundance. Model 4 and Model 5 were developed under assumption that there may be lagged relationships between hydroclimatic processes and mosquito abundance in the region. While the performance of models are better than the individual process based models (Model 1 and Model 2), but is lower than Model 3. It also suggests and strengthens the argument that both LST and temperature are the contributing factors for WNV mosquito abundance.

**Table 2:** **Model performance statistics based on binomial logistical regression analysis**.

	Model 1	Model 2	Model 3	Model 4	Model 5
Goodman–Kruskal Gamma	0.39	0.42	0.65	0.54	0.48
Hosmer–Lemeshow (df: 8) – *p*-value	0.63	0.60	0.75	0.63	0.55
Kendall Tau-a	0.14	0.24	0.60	0.31	0.45
Pearson *p*-value	0.61	0.62	0.64	0.55	0.57

$P\left(Y\right)=\frac{\exp\;(Model_{x})}{\left(1+\exp\;(Model_{x})\right)}$.

*Model_1_ = α_1_ + β_2_. LST*.

*Model_2_ = α_2_ + β_2_. Precipitation*.

*Model_3_ = α_3_ + β_3_. LST + γ_3_. Precipitation*.

*Model_4_ = α_4_ + β_4_. LST*_t−_*_1_ + γ_4_. Precipitation*_t−_*_1_*.

*Model_5_ = α_5_ + β_5_. LST*_t_*_−1_ + γ_5_. Precipitation*_t_**.

*Goodman–Kruskal Gamma, Hosmer–Lemeshow, Kendall Tau-a, Pearson varies between 0 and 1; α*_x_*, β*_x_*, γ*_x_* are model coefficients. Monthly data from 2003 to 2012 were used to develop the model. Logistical regression models were preferred [based on usability from literature Brown et al. (16)] since the exact number of WNV positive mosquitoes could not be ascertained, but probability of occurrence of WNV mosquitoes can be estimated*.

Warm temperature was reported (17) to increase mosquito population numbers and subsequent human infections with the disease agent transmitted by mosquitoes. The basic mechanism by which temperature has been related to mosquito growth is the decrease caused in the length of the gonotrophic cycle, shortening the extrinsic incubation period of the virus its vector, thereby enhancing growth of the virus (18, 19). The suggested relationship of the impact of vegetation is consistent with results of several studies associating vector-borne mosquito infections (16, 20, 21). However, in our modeling experiments, NDVI was not statistically significant and hence were not used. This is consistent with other studies (1), since regions where NDVI values are saturated (>0.85), inter-annual variability may not be sufficient to explain association of mosquito population with NDVI. Role of precipitation in creating localized favorable conditions for WNV mosquitoes is reported in literature (19, 22), therefore, results from our study is likely to be applicable to other regions of similar geomorphology.

## Conclusion

Hydroclimatic processes measured by satellite remote sensing can be useful in identifying conditions critical to emergence of the vector-borne WNV and the outbreak of human disease that occurred in a historically disease free region. Changes in LST, and regional precipitation provided a favorable environment for the mosquitoes. Using MODIS derived LST for warm temperature, the observed increase in temperature, in combination with already present vegetation and heavy seasonal rainfall, is concluded to be associated with the an increase in the mosquito population in southwestern West Virginia.

Nonetheless, the study has a few limitations posed by the availability of satellite data. MODIS data are available as a snapshot due to sun synchronous orbit; therefore, daily average temperature cannot be estimated. However, the contextual information provided by such data is able to characterize hydroclimatic variability in the large-scale geophysical processes and hence, it is a valuable resource for possible disease surveillance. Monitoring mosquito populations by satellite based hydroclimatic processes has been suggested by other investigators, but the key contribution of our study is the diagnostic understanding, based on large-scale hydroclimatic processes measured by remote sensing during areal surveillance and prediction of mosquito abundance, to identify risk zones is unique. Both abundance of mosquito-borne arboviruses, including WNV, and data on related human infections are required to determine space-time evolution of the disease. Disease surveillance data cannot easily be gathered on large geographical scales and this causes a scale mismatch between the massive data gathered on geophysical processes versus that for disease agent and its vector. Utilization of intervention strategies, such as chemical spraying to reduce mosquito populations, has had a contrary effect, i.e., solution for virulent pathogen and adapted insect populations. In contrast, satellite monitoring of those hydroclimatic processes that have been linked to mosquito abundance offers a useful means of developing predictive tools.

## Conflict of Interest Statement

The authors declare that the research was conducted in the absence of any commercial or financial relationships that could be construed as a potential conflict of interest.
